# Child Welfare at Brussels

**Published:** 1921-07-30

**Authors:** 


### THE OBLIGATION OF CONDUCT.

### Child Welfare at Brussels.

**By a SOCIAL WORKER.**

It is probably only by slow degrees that most people in European countries will come to understand the significance of the founding in Brussels of a permanent child-welfare office. Here activities on behalf of children will centre, and thence efforts directed by the newest enlightenment are intended to diverge. If we may use a hard-worked and often ill-used word, it is an epoch-making event.

It marks, for one thing, the crystallising into the concrete form necessary for action of those thoughts about childhood which affect conduct towards them and which are new to this age, and a reaction from a very different mode of thought. In that now discredited view there were blended the remains of many old ideas which in their turn have ruled the world. At one time the child has been chiefly the chattel, liable to be sold for gain or to be offered in sacrifice to deities; at another he has been a bread-winner; at another he has been a soul in peril, needing iron training and ceaseless chastisement to save him from surrounding and inherent evil; at another, by reaction especially from that gloomy notion, he has been pet and plaything, the subject of joke and idyllic romance. Now, at last, the truly Christian view conquers, and shows him as a budding life, a gift of God to be welcomed with thanksgiving and tended with care, helped and watched over as a garden plant till strong enough in fibre to withstand storm and come to due fruition.

It is natural, therefore, that, as the medical correspondent of *The Times* especially notes, the present step is a challenge not merely to the world to do its utmost for the child, but also to the child to do its best for the world. "Wherefore, when I looked that it should have brought forth grapes, brought it forth wild grapes?"

Well, why? Were training and restraining given in due season and due measure? Was the child received in a fit home where the conditions, however simple, were healthful to mind and body? We are not surprised to learn that the illegitimate child is to be a special subject of study.

It will be a great step in advance if the child, whenever possible, is given the father's name. The more his responsibility can be brought home, the better hope there will be of lessening the terrible death-rate of 155 to 1,000 births in contrast with the average eighty of legitimates. But safeguards will be needed against blackmail, and assistance towards the protection of very young girls, forcible if necessary. There is no more alarming fact known to social workers than the present ready evil-doing of girls who are mere children to obtain money for smart clothing and sweets. The careful study of wise, as distinguished from some very unwise, sex education will be needed to attack this trouble at its very roots. Probably the schoolmaster was right who lately said that at about seventeen all boys should be clearly told the social results of prostitution in the past and present. What a pitiful anomaly a lad might see it to be that, while his mother and sisters were working to support some rescue home, he and his brothers were helping to supply it with inmates!

The abnormal and the criminal child are naturally as important a subject of study. Much is being done, much remains to do. The present writer confesses to one fear—that of danger in too much classifying as abnormal. Children pass through many phases: sometimes a quiet, well-behaved child will have an attack of something like moral measles; it may be violent temper, or untruthfulness, or some odd tendency. A young lady who grew up perfectly normal once related to the writer that she suffered for several childish years from a frantic desire to eat live flies, the larger and more loudly buzzing the better. Shame concealed the craze, which one day harmlessly disappeared. But in days when the elders are less unapproachable such lesser abnormalities will come more easily into daylight, which is the first step to a cure. Children and young people have too much discussed among themselves matters on which they needed guidance, and so have come to wrong conclusions. In France girls especially have had less opportunity of exchanging long confidences. But the removal for observation of a really abnormal child, if carefully carried out, and if no label is affixed unless absolutely necessary to the one so watched, will save much harmful suggestion within the family.

Above all, let us not try to keep the child "a child," because life has cares. Children love responsibility, and is not much of the lamented folly of youth attributable to the very fact that parents expect "a sense of responsibility" to grow with nothing to feed on? Let the young people look on life as a great adventure. Let them manage something as soon as possible and not be managed, and managed for, until grown up, possibly even until growing old. Then we may more hopefully look for a return from them for all that is done, and for their service, early begun, to the generation after next, and so for real progress.

